# Validation for using electronic health records to identify community acquired pneumonia hospitalization among people with and without HIV

**DOI:** 10.1186/s41479-020-00068-1

**Published:** 2020-07-25

**Authors:** Maria C. Rodriguez-Barradas, Kathleen A. McGinnis, Kathleen Akgün, Janet P. Tate, Sheldon T. Brown, Adeel A. Butt, Michael Fine, Matthew Bidwell Goetz, Christopher J. Graber, Laurence Huang, David Rimland, Amy Justice, Kristina Crothers

**Affiliations:** 1 Michael E. DeBakey VAMC and Baylor College of Medicine, Houston, TX USA; 2 VA Connecticut Healthcare System, West Haven, CT USA; 3 Yale University, New Haven, CT USA; 4 James J Peters VAMC, Bronx, NY USA; 5 Icahn School of Medicine at Mount Sinai, New York, NY USA; 6 VA Pittsburgh Healthcare System, Pittsburgh, PA USA; 7 Weill Cornell Medical College, New York, NY USA; 8 Weill Cornell Medical College, Doha, Qatar; 9 VA Greater Los Angeles Healthcare System, and David Geffen School of Medicine at UCLA, Los Angeles, CA USA; 10 San Francisco General Hospital and University of California San Francisco, San Francisco, CA USA; 11 VAMC and Emory University School of Medicine, Atlanta, GA USA; 12 University of Washington, Seattle, WA USA

**Keywords:** Community-acquired pneumonia, HIV, Electronic health records

## Abstract

**Background:**

Cohort studies identifying the incidence, complications and co-morbidities associated with community acquired pneumonia (CAP) are largely based on administrative datasets and rely on International Classification of Diseases (ICD) codes; however, the reliability of ICD codes for hospital admissions for CAP in people with HIV (PWH) has not been systematically assessed.

**Methods:**

We used data from the Veterans Aging Cohort Study survey sample (*N* = 6824; 3410 PWH and 3414 uninfected) to validate the use of electronic health records (EHR) data to identify CAP hospitalizations when compared to chart review and to compare the performance in PWH vs. uninfected patients. We used different EHR algorithms that included a broad set of CAP ICD-9 codes, a set restricted to bacterial and viral CAP codes, and algorithms that included pharmacy data and/or other ICD-9 diagnoses frequently associated with CAP. We also compared microbiologic workup and etiologic diagnosis by HIV status among those with CAP.

**Results:**

Five hundred forty-nine patients were identified as having an ICD-9 code compatible with a CAP diagnosis (13% of PWH and 4% of the uninfected, *p* < 0.01). The EHR algorithm with the best overall positive predictive value (82%) was obtained by using the restricted set of ICD-9 codes (480–487) in primary position or secondary only to selected codes as primary (HIV disease, respiratory failure, sepsis or bacteremia) with the addition of EHR pharmacy data; this algorithm yielded PPVs of 83% in PWH and 73% in uninfected (*P* = 0.1) groups. Adding aspiration pneumonia (ICD-9 code 507) to any of the ICD-9 code/pharmacy combinations increased the number of cases but decreased the overall PPV. Allowing COPD exacerbation in the primary position improved the PPV among the uninfected group only (to 76%). More PWH than uninfected patients underwent microbiologic evaluation or had respiratory samples submitted.

**Conclusions:**

ICD-9 code-based algorithms perform similarly to identify CAP in PLWH and uninfected individuals. Adding antimicrobial use data and allowing as primary diagnoses ICD-9 codes frequently used in patients with CAP improved the performance of the algorithms in both groups of patients. The algorithms consistently performed better among PWH.

## Background

Community acquired pneumonia (CAP) remains one of the most frequent complications in people with HIV (PWH) as well as in the aging population [1–3]. Despite widespread use of antiretroviral therapy (ART), incidence of CAP continues to be higher in PWH compared to HIV-uninfected (uninfected) counterparts [1, 2]. As the proportion of older PWH increases, it is important to identify similarities and differences in risk factors for and presentation of CAP between older people with and without HIV [1, 2].

Cohort studies identifying the incidence, complications and co-morbidities associated with CAP are largely based on administrative datasets from hospitals and/or research networks [1, 4–6]. The majority of these studies rely on International Classification of Diseases (ICD) codes but their performance is seldomly validated [6–9]. One caveat with the use of ICD pneumonia codes is that they do not distinguish between community-acquired and hospital-acquired disease [10, 11]; and ICD codes often need to be combined with other means for identifying patients with CAP among the patient population included in administrative datasets [7, 11]. Administrative datasets are also used for studies evaluating CAP outcomes among PWH; however, the reliability of ICD codes for hospital admissions for CAP in PWH compared to those uninfected has not been systematically assessed [1, 12]. Hence, it is important to understand and validate their performance amongst PWH [12, 13]. Cohort studies specifically assessing the performance of ICD-9 codes in identifying the patients admitted with CAP have either excluded PWH [14], excluded those with lower CD4 cell count [6], PWH represented a very small percentage of the patient population in their sample [15], or there is no mention of HIV at all [16].

In order to address the above issues, we used data from the Veterans Aging Cohort Study (VACS) survey sample to 1) determine if there were differences in the clinical characteristics, microbiologic workup and etiologic diagnosis in PWH compared to uninfected patients with clinically confirmed CAP based on chart review, and 2) validate the use of electronic health records (EHR) data in conjunction with ICD-9 codes to identify CAP hospitalizations with the greatest accuracy when compared to chart review.

## Methods

The VACS survey cohort consists of PWH and site, age, sex, and race/ethnicity matched uninfected patients at eight Veterans Health Administration facilities in Atlanta, Baltimore, Houston, Los Angeles, Manhattan, Bronx, Pittsburgh, and Washington, DC and is described in more detail elsewhere [17]. To identify patients with a potential CAP hospitalization, we searched the EHR of the 6824 subjects in VACS survey cohort (3410 PWH, 3414 uninfected) with baseline enrollment date from June 2002 to July 2008 for the first inpatient ICD 9th classification of diseases codes (ICD-9 codes) consistent with possible CAP after VACS enrollment and up until September 2008. We included codes typically used for CAP such as bacterial (481–486) or viral pneumonia (480, 487) (restricted CAP codes), and additional ICD-9 codes, consistent with miscellaneous lung infections, including aspiration pneumonitis (507), inhalation (506), empyema (510), pleurisy (511.1), and lung abscess (513) and other infections associated with pneumonia and/or due to infection elsewhere, (3.22, 21.2, 39.1, 52.1, 55.1, 73, 517.1) (broad set of codes). ICD-9 code groupings assessed were based on those most commonly used in published studies [11, 14, 18, 19]; restricted plus broad set of codes were chosen to capture the largest potential number of pneumonia events. Those patients with inpatient codes for fungal (including *Pneumocystis jiroveci*) or mycobacterial etiologies of pneumonia but none of the qualifying CAP codes were excluded. Only the first CAP event for each patient was included.

Demographic, clinical, and laboratory data were retrieved from the VACS database. Baseline date was defined as that corresponding to a patient’s enrollment in VACS survey cohort. Behaviors and comorbidities of interest were those known to be associated with increased risk for pneumonia. Smoking, hazardous alcohol use and injection drug use (IDU) were identified using self-reported survey data. Hazardous alcohol use was based on having an Alcohol Use Disorders Identification Test (AUDIT-C) score of ≥4 for men/≥3 for women. Diabetes mellitus and chronic obstructive pulmonary disease (COPD), were defined as presence of one ICD-9 inpatient diagnosis code or 2 or more outpatient diagnoses codes, and/or by laboratory and/or pharmacy data [i.e., for diabetes mellitus, HbA1c results or prescription of antidiabetic medications] [13]. Chronic kidney disease (CKD) was defined as EGFR < 60 cc/min. Hepatitis C virus (HCV) infection was defined using a combination of ICD-9 codes and HCV antibody and HCV RNA laboratory results.

### CAP confirmed by chart review

At each site, the EHR of patients identified by the broad set of potential CAP ICD-9 codes (listed above) were reviewed by site principal investigators using standardized forms (see Additional file 1). CAP was considered to be present on admission when all three of the following criteria were met on chart review: a) clinical findings consistent with CAP: symptoms (e.g. new cough, sputum, lethargy, fever), signs (tachycardia, tachypnea, findings on chest exam), and/or laboratory abnormalities (leukocytosis, leukopenia); b) chest radiologic findings consistent with CAP (localized or diffuse infiltrate(s), consolidation, pleural effusion) as assessed in the clinical radiology report; and c) receipt of antibacterial drugs recommended by guidelines for treatment of CAP within 48 h of admission. Those fulfilling these three criteria were categorized as confirmed CAP present on admission per the gold-standard of chart review (confirmed-CAP). Patients with confirmed-CAP were assessed for risk factors for infections with antimicrobial-resistant organisms. Risk factors included discharge from acute care hospital within 90 days of admission with CAP, hemodialysis, IV antibiotics or chemotherapy, wound care within 30 days, or transfer from a nursing home (NH). Patients transferred from another health-care facility were included in the analysis if their initial presentation was for a diagnosis consistent with CAP. Patients who developed pneumonia > 48 h after admission to the VA as determined by chart review were categorized as hospital-acquired pneumonia (HAP) and were excluded from further analyses. Microbiologic diagnosis of confirmed-CAP was categorized as definitive (likely pathogen isolated from sterile site or positive antigen test), presumed (identification of likely pathogen from sputum or other respiratory sample), or suspected (all others), by data abstracted from chart review.

### Comparing ICD-9 codes, with or without receipt of antimicrobial medication, against chart review for identifying CAP

We calculated CAP frequency, incidence, and PWH to uninfected rate ratios for confirmed-CAP, for the initial broad set of inpatient ICD-9 codes (previously listed) and for different smaller subsets of ICD-9 codes. These codes were allowed in any position to be able to capture all patients with a potential diagnosis of CAP. We also explored algorithms including pharmacy data obtained from the EHR; we chose as the pharmacy parameter antimicrobial prescriptions ≤72 h after admission (instead of the within 48 h criteria used for chart review) to account to fact that within VA EHR, antibiotics prescribed in emergency department may not be reflected in the inpatient pharmacy data and for the use of antibiotics with 24 h dosing frequently used as initial therapy (i.e.; ceftriaxone, azithromycin, levofloxacin) [20–22]. We also explored the performance of algorithms where the ICD-9 code for CAP was required to be in the primary position or secondary only to select diagnoses frequently used as primary diagnosis on patients admitted with CAP [sepsis (995.91, 995.92), bacteremia (790.70) or respiratory failure (518.81, 518.82, 518.85)]. This strategy has been utilized in other administrative data-based studies and has the objective of enhancing the search towards patients with high likelihood of having CAP on admission [15, 23, 24].

We explored data within the national VACS cohort of over 50,000 PWH and 100,000 uninfected patients [25] to inform which other codes to evaluate in the primary position. In addition to sepsis and respiratory failure, we identified chronic bronchitis with acute exacerbation (491.2) and congestive heart failure (428) as among the most common primary codes in the national VACS. Not surprisingly, among PWH, HIV was the most common primary ICD-9 code with secondary code of CAP. Therefore, we evaluated algorithms that included HIV, sepsis, bacteremia, respiratory failure, chronic bronchitis with acute exacerbation and congestive heart failure as primary diagnoses with pneumonia ICD-9 codes in secondary diagnosis position.

### Statistical analyses

Characteristics of the VACS patients and of those with confirmed-CAP were described and compared by HIV status using chi-square tests for categorical variables and student’s T-test or Wilcoxon Rank-sum for normally or non-normally distributed variables. Of the 6824 patients enrolled in the VACS survey cohort, we generated HIV stratified frequencies, incidence rates, and hazard rate ratios from Cox Proportional Hazard models adjusted for age, race/ethnicity, and sex for the outcomes of confirmed-CAP as well as for the different ICD-9 code algorithms to identify CAP. These algorithms consisted of varying the specific CAP ICD-9 codes included and their position, with or without antibiotics within 72 h of admission. The different ICD-9 code groupings used included: a) the broad set of CAP-related ICD-9 codes for which we initially searched, b) restricted CAP ICD-9 codes in any position (algorithm 1), and c) restricted CAP ICD-9 codes in primary position or secondary only to HIV, sepsis, bacteremia, and respiratory failure with or without chronic bronchitis with acute exacerbation (491.2) and congestive heart failure with acute exacerbation (428) in primary position (algorithm 2). To determine which set of ICD-9 codes performed best based on positive predictive value (PPV), we calculated the percent of subjects with confirmed-CAP of those identified with each ICD-9 code algorithm and antimicrobial subset, if applicable. PPVs were calculated overall and by HIV status; *p*-values from chi-square tests were used to compare PPVs between PWH and those uninfected. Sensitivity and specificity could not be calculated because chart review was performed only on those 549 with an initial CAP-related diagnosis by the broad set of ICD-9 codes and not on the 6824 patients in this VACS cohort. Analyses were carried out using Stata 14.2 (College Station, TX).

## Results

### Patients with ICD-9 codes compatible with CAP diagnosis

Of the 6824 patients enrolled in VACS, 3410 were PWH and 3414 were uninfected. The mean age for PWH and those uninfected, respectively, was 49 years and 50 years. The majority were African American (67% vs. 63%), and male (97% vs. 92%). Mean observation time was 4.3 years (SD 1.8) and 4.3 years (SD 1.5), respectively.

The prevalence of behaviors and comorbidities associated with increased risk for CAP were unevenly distributed by HIV status (Table 1). The prevalence of ever smoking, history of drug use, and hepatitis C infection were higher in the PWH compared to those uninfected (77% vs. 72, 33% vs. 16%, and 53 vs. 28%, respectively [all *p* < 0.01]), while the prevalence of diabetes mellitus and hazardous alcohol use was higher in the uninfected compared to PWH (38% vs. 28 and 28% vs. 26%, respectively [*P* < 0.01]).

**Table 1:** Characteristics at baseline of VACS Patients enrolled in survey sample and of those with clinically confirmed community acquired pneumonia (confirmed-CAP) within the sample

	VACS Survey Sample				With Confirmed-CAP			
	Total	PWH	Uninfected	***P***	Total	PWH	Uninfected	***P***
**Demographics**	6824	3410	3414	–	397	321	76	–
Mean age in years (SD)	49.8 (9.4)	49.1 (8.8)	50.4 (10.0)	**< 0.001**	53.3	52.2	57.8	**< 0.001**
Male Gender	95%	97%	92%	**< 0.001**	97%	98%	95%	0.1
Non-white race	79%	81%	76%	**< 0.001**	86%	89%	72%	**0.002**
**Behaviors**								
Tobacco use ever	75%	77%	72%	**< 0.001**	86%	86%	88%	0.3
Hazardous alcohol use	27%	26%	28%	**0.009**	29%	29%	28%	0.8
IV drug use ever	25%	33%	16%	**< 0.001**	41%	47%	16%	**< 0.001**
**Comorbidities**								
Diabetes mellitus	33%	28%	38%	**< 0.001**	44%	41%	54%	**.04**
CKD	12%	13%	11%	.07	24%	22%	28%	0.4
COPD	5%	5%	5%	.7	10%	9%	14%	0.2
HCV	41%	53%	28%	**< 0.001**	58%	65%	28%	**< 0.001**
**HIV Parameters**								
CD4 ≥ 200 cells/μL	–	76%	–	–	–	64%	–	–
On antiretroviral treatment	–	72%	–	–	–	68%	–	–
VL ≤500 RNA copies/mL	–	50%	–	–	–	40%	–	–
**EHR CAP Codes**								
Any Inpatient CAP ICD-9 code, n (%)	549 (8)	433 (13)	116 (4)	**< 0.001**	–	–	–	–
Confirmed-CAP by chart review, n (%)	397 (6)	321 (9)	76 (2)	**< 0.001**	**–**	**–**	**–**	**–**

Age, at time of VACS survey for VACS survey sample, age at time of CAP for those with confirmed-CAP; hazardous alcohol use, based on AUDIT-C score EGFR; IV intravenous; *CKD* Chronic kidney disease defined as estimated glomerular filtration rate < 60 ml/min, *COPD* Chronic obstructive pulmonary disease, *HCV* Hepatitis C infection based on antibody test results and/or HCV RNA, *CD4* CD4 cell count, *VL* Viral load, *EHR* Electronic health record

Of the 6824 patients, 549 (8%) were identified as having at least 1 inpatient ICD-9 code compatible with a possible CAP diagnosis (13% of the PWH and 4% of the uninfected groups, *p* < 0.001) (Table 1). Chart review of these 549 patients revealed that there were no differences between PWH and uninfected groups in the presence of the individual criteria for CAP: clinical signs or symptoms (93% vs. 92%), radiologic findings (76% vs. 67%), and antibacterial use (89% vs. 87%) (*P* ≥ 0.1 for all comparisons) (Table 2). However, a non-statistically significantly higher rate of PWH fulfilled all three criteria for confirmed-CAP, 74% vs. 66%, respectively (*P* = 0.2). In 107 subjects with ICD-9 codes for CAP but for whom CAP on admission could not be confirmed by chart review (77 PWH, 30 uninfected), three quarters met clinical criteria for CAP (72 and 77%, respectively), and more than half received antibacterials targeting CAP within 48 h of admission (62 and 63%, respectively). However, only 8 and 7%, respectively, met radiologic criteria for pneumonia, (*P* ≥ 0.1). The proportion of those not fulfilling all three criteria for confirmed-CAP, those categorized as hospital-acquired pneumonia, or those for whom there was not enough information to adjudicate a definitive diagnosis was similar by HIV status (Table 2).

**Table 2:** Subjects admitted with community-acquired pneumonia (CAP) identified by ICD-9 codes. CAP ICD-9 codes are those consistent with bacterial or viral pneumonia. Confirmed-CAP present on admission are those that fulfill the three established criteria (A-clinical, B-radiologic, and C-antimicrobial for CAP) by chart review

	Total 549 N (%)	PWH 433 N (%)	Uninfected 116 N (%)	***P***
**Confirmed-CAP (A, B and C present)**	397 (72)	321 (74)	76 (66)	0.2
A. Clinical criteria present	508 (93)	401 (93)	107 (92)	0.7
B. Radiologic criteria present	407 (74)	329 (76)	78 (67)	0.1
C. Antimicrobials for CAP	489 (89)	387 (89)	102 (87)	0.6
**CAP Not confirmed (A, B or C missing)**	107 (20)	77 (18)	30 (26)	0.4
A. Clinical criteria present	79 (73)	56 (72)	23 (77)	0.6
B. Radiologic criteria present	8 (7)	6 (8)	2 (7)	0.9
C. Antimicrobials for CAP	67 (62)	48 (62)	19 (63)	0.9
**Hospital-acquired Pneumonia (HAP)**	14 (3)	9 (2)	5 (4)	0.8
**Unable to adjudicate**	31 (6)	26 (6)	5 (4)	0.2

A-Clinical, symptoms, signs and/or laboratory finding compatible with CAP

B-Radiologic, chest radiograph and/or CT chest compatible with CAP

C-Antimicrobials for CAP, antimicrobials administered within 48 h of admission

### Clinical characteristics of patients with confirmed-CAP

Of the 397 subjects with confirmed-CAP, 321 PWH and 76 uninfected, mean age at time of CAP diagnosis was 52 and 58 years (*p* < 0.001), respectively. Similar to the VACS overall, the majority were non-White (89% vs. 72%, *p* = 0.002) and male (98% vs. 95%, *p* = 0.1) (Table 1).

Overall and by HIV status, compared to the VACS survey sample without CAP, confirmed-CAP patients were more likely to have ever smoked, and to have a diagnosis of diabetes mellitus, COPD, and CKD (all *P* < 0.05). Among the confirmed-CAP subjects, compared to the uninfected group, PWH were younger and more likely to have history of IDU and diagnosis of hepatitis C, while the uninfected were more likely to have diagnosis of diabetes mellitus; the rates of smoking and other comorbidities were similar. Of note, hazardous alcohol use was not more prevalent among CAP patients compared to those without CAP (29% vs. 27%, *P* = 0.4), and among those with CAP, was not different between PWH and those uninfected (29% vs. 28%, *P* = 0.8). Among PWH with confirmed-CAP, the majority had CD4 cell count > 200/mm^3^ (64%), were on antiretroviral therapy (68%); and 40% had non-detectable viral load (< 500 RNA copies/mm^3^); these rates were, however, lower than those of the survey sample without CAP (77%, *p* < .001; 72%, *p* = .12; and 51%, *p* < .001 respectively; otherwise not shown).

Although the majority of the patients with confirmed-CAP (89%) were admitted from home and based on exposures (as described under methods section) had no identifiable risk factors associated with infection with multi-drug resistant organisms, significantly more subjects in the uninfected group (19%) had risk factors for infections with resistant organisms compared to PWH (9%) (*P* = 0.04); this difference was driven mostly by more uninfected patients being transferred to the hospital from skilled nursing or other non-acute health care facilities (11% vs. 3%, respectively).

### Etiologic diagnosis of confirmed-CAP

More PWH (93%) than uninfected (82%) underwent any microbiologic evaluation for bacterial detection (blood and/or respiratory sample submitted for culture) within 48 h of admission (*P* < 0.01). Similarly, more PWH had respiratory samples submitted than those uninfected (64% vs 41%, *p* < 0.05). Despite the relatively high percentage of subjects with samples submitted for evaluation, a definitive microbiological diagnosis of bacterial pneumonia was only achieved for 11% of the patients (13% of PWH, 5% of uninfected, *P* = 0.14). In the majority of the patients in both groups (77% in PWH and 84% in uninfected) the diagnosis could only be classified as suspected (criteria for definitive or presumed not met).

### Performance of ICD-9 codes and pharmacy data algorithms for CAP

Table 3 shows the PPVs for confirmed-CAP for the different ICD-9 groupings with or without the EHR pharmacy data on antibacterial use within 72 h of admission, and for subsets of pneumonia ICD-9 codes in any position versus as primary codes and secondary only to HIV, respiratory failure, bacteremia, or sepsis (codes frequently listed as primary in patients admitted with CAP), as well as chronic bronchitis and congestive heart failure (conditions found within VA to be frequently listed as primary in patients with CAP). Overall, the PPV of the initial broad set of all inpatient ICD-9 codes was 72%, not statistically significantly higher in the PWH (74%) compared to the uninfected (66%) (*P* = 0.07) and was modestly enhanced with the addition of the pharmacy data. The best overall PPV (82%) was obtained by using the restricted set of ICD-9 codes (480–487) in primary position or secondary only to selected codes as primary (HIV disease, respiratory failure, sepsis or bacteremia) with the addition of EHR pharmacy data; this algorithm yielded PPVs of 83% in PWH and 73% in uninfected (*P* = 0.1) groups. The next best overall PPV (81%) did not include pharmacy data and was obtained by using a reduced set of ICD-9 codes [480–487] in primary position and adding COPD with acute exacerbation (491) to the selected codes (HIV disease, respiratory failure, sepsis or bacteremia) for which the pneumonia codes could be secondary diagnosis; this algorithm yielded PPVs of 82% for PWH and 76% for uninfected (*P* = 0.3). Adding aspiration pneumonia (ICD-9 code 507) to any of the ICD-9 code/pharmacy combinations increased the number of cases but decreased the PPV. The results for other algorithms explored are shown in Table 3.

**Table 3:** Incidence Rates (IR) and Hazard Ratios (HR) for community-acquired pneumonia (CAP) identified in electronic health records (EHR) by ICD-9 codes and restricted to those with confirmed CAP by chart review (confirmed-CAP) in PWH and HIV-uninfected (Uninf) subjects

	In VACS Survey Cohort							Of those initially identified as having an EHR CAP-related code					
	Frequency		IR		PWH to Uninfected					PPV			
	PWH ***N*** = 3410	Uninf ***N*** = 3414	PWH	Uninf	HR	P	95% CI	Total	Confirmed-CAP	Total	PWH	Uninf	p-value
**All codes**	433	116	2.98	0.70	4.25	<.001	3.45–5.22	**549**	**397**	72%	74%	66%	0.065
All codes plus Abx	415	104	2.84	0.63	4.54	<.001	3.65–5.64	519	386	74%	76%	69%	0.18
**Algorithm 1, 480–487 in any position**	413	98	2.83	0.59	4.75	<.001	3.81–5.94	511	380	74%	76%	69%	0.21
Algorithm 1 plus Abx	398	88	2.72	0.53	5.10	<.001	4.04–6.44	486	369	76%	77%	73%	0.47
Algorithm 1, plus 507 in any position	426	114	2.92	0.69	4.22	<.001	3.43–5.21	540	393	73%	75%	66%	0.059
Algorithm 1, plus 507 in any position plus Abx	409	102	2.80	0.62	4.53	<.001	3.64–5.64	511	382	75%	76%	70%	0.181
**Algorithm 2, 480–487 in primary or secondary position only to selected diagnosis**^**a**^	329	59	2.24	0.36	6.22	<.001	4.71–8.24	388	313	81%	82%	75%	0.2
Algorithm 2 plus abx	317	57	2.15	0.34	6.22	<.001	4.68–8.27	374	305	82%	83%	73%	0.096
Algorithm 2 plus 507	334	69	2.27	0.42	5.42	<.001	4.17–7.04	403	319	79%	81%	70%	0.031
Algorithm 2 plus 428	331	62	2.25	0.37	5.96	<.001	4.53–7.83	393	315	80%	82%	71%	0.048
Algorithm 2 plus 491	334	63	2.27	0.38	5.94	<.001	4.53–7.80	397	321	81%	82%	76%	0.3
Algorithm 2 plus 507, plus 428 and plus 491	341	76	2.32	0.46	5.03	<.001	3.91–6.47	417	341	79%	81%	68%	0.013
**Confirmed-CAP**	321	76	2.18	0.46	4.73	<.001	3.67–6.08	na	na	Na	na	na	na

HR, adjusted for age, race/ethnicity, and gender

All codes, refers to the initial set of CAP related ICD-9 codes used listed in methods

Plus Abx, refers to restricting to those that received antimicrobials within 72 h of admission identified by pharmacy package in EHR

480–487 refers to the set of restricted CAP codes

Plus 507 refers to allowing the aspiration code with the main set of CAP codes

^**a**^CAP-related codes in primary position with the exception that they could be in secondary position if the following HIV, sepsis, respiratory insufficiency, and bacteremia codes were in primary position: 042, 038, 518.81, 518.82, 518.85, 995.91, 995.92, 790.7, 790.70

Plus 428 refers to adding the heart failure code to the non-CAP related codes allowed in primary position

Plus 491 refers to adding the COPD code to the non-CAP related codes allowed in primary position

## Discussion

In this study we found that a) in the ART era, Veterans living with HIV remain at increased risk for presenting with CAP requiring hospitalization and, b) ICD-9 code-based algorithms perform similarly to identify CAP in PWH and uninfected patients. Specifically, within the VACS survey cohort, significantly more PWH subjects (13% PWH vs. 4% uninfected) had an ICD-9 code for CAP; and in 74 and 66%, respectively, the diagnosis was confirmed by chart review (9.4 and 2.3%, respectively of the total sample). Among those with CAP, PWH were younger and more likely to be non-White and to have history of IDU, and consequently, had higher prevalence of hepatitis C infection compared to those without HIV. In accordance with prior studies of PWH [1, 25], those with CAP were less likely to be on ART and more likely to have lower CD4 cell count and detectable viral load compared to those without CAP [26, 27]. The uninfected group had a significantly higher prevalence of diabetes mellitus in the survey sample (38%) and CAP-confirmed sample (54%) compared to the PWH; however, similarly as the uninfected, significantly more PWH with CAP had diabetes mellitus (41%) compared to PWH in survey sample (28%) (*P* < 0.01), suggesting that diabetes adds to risk for pneumonia in both groups.

Although significantly more PWH had samples submitted for microbiologic evaluation, the overall diagnostic yield was low and not significantly different between the groups. Consistent with recent studies [28–30], for the majority of patients admitted with pneumonia, no microbiologic etiology was identified despite the wide use of diagnostic tools for mostly bacterial etiologies. The proportion of patients with identifiable bacterial causes for CAP has not changed as newer diagnostic tools (PCR-based diagnositics) have been incorporated into clinical testing algorithms; on the contrary, in the USA, the rate has been consistently low [31].

The overall PPV for ICD-9 codes for pneumonia was 72% and was not significantly different between the two groups. PPV was equally enhanced by either using a subset of more specific ICD-9 codes for pneumonia or by adding antimicrobial use within 72 h of admission to the broad, all encompassing, set of codes; the best overall PPV (82%) was obtained with the use of the restricted set of ICD-9 codes in primary position or secondary only to selected codes as primary with the addition of antimicrobial use. For the time period that this study encompasses, molecular testing for viral etiologies was not widely available. It is possible that establishing specific viral diagnosis may enhance the PPV for ICD code algorithms that include viral causes for pneumonias [32].

Many studies have tried to assess and validate the use of ICD-9 codes to accurately identify CAP admissions (Table 4). These studies have mostly used what we referred to as the restricted CAP codes (480–487), some have added algorithms with antimicrobial use [7], or have included a combination of databases in their algorithms [11, 14, 15]. Only one study has specifically validated the accuracy of the codes in PWH [13] and none has compared its accuracy among PWH and uninfected groups within the same cohort. Among these studies, the PPV for ICD-9 codes has ranged from 57% [20] to as high as 97% [24]. The range of PPV obtained with the different code combinations and algorithms in our study (72–82%) was very similar to that of studies that used similar approach [11]. In contrast to Aronsky et al. study [11], in our study, adding aspiration pneumonia to the algorithm decreased the PPV. Including aspiration pneumonia in CAP studies remains controversial as some of those cases may not represent infection [33]. The reason for the variable range of PPV among different studies may have to do with the way the cohort is selected, the coding practices of the different health care systems, and the criteria used for validation, among other reasons [34].

**Table 4:** Positive predictive value (PPV) for pneumonia identified by ICD codes

Reference	Study years	Cohort size	Databases	ICD-Codes	Included HIV subjects	Gold standard	Chart confirmed pneumonia	Sensitivity/ Specificity	PPV
Marrie [18]	1984	127	ICD-9			Research chart review			57%
Whittle [14]	1989–1990	212	PMC + ICD-9 DRG	PMC + ICD-9 Algorithms: #1 Primary or secondary to related codes #2 Only Primary	Excluded	Chart review	65% CAP 24% HAP 11% no pneumonia	#1 89%/80% #2 84%/86%	#1 89% #2 92%
Draper [16]	1990	4381	ICD-9	ICD-9 codes for pneumonia any position		Chart review	27% no pneumonia		
Justice [13]	1999–2000	881			Only PWH	Chart review *N* = 27		53%/95%	
Aronsky [11]	1999–2000	10,748	ICD-9 DRG	Algorithms #1 restricted codes #2 + 507 and others #3 + resp. failure/sepsis	Not mentioned	Chart review	*N* = 272	#1 55%/99% #2 65%/99.6%	#1 79% #2 81% #3 80%
Yu [19]	1997–2005	> 10,000	ICD-9	Algorithms #1480,487 or 507 as primary #2 Codes any position + LOS + age + other codes	Not mentioned	Chart review *N* = 3991	62% CAP Results categorized by age group (18–64, and ≥ 65 years).	#1 18–64 63%/93% ≥65 65%/85%	#1 18–64, 91% ≥65, 89% #2 18–64, 84% ≥65, 82%
Drahos [7]	2000–2010	25,064	ICD9–9 Pharmacy data	Algorithms #1480–486 #2 codes + verified abx script	Not mentioned	Chart review *N* = 175	88% pneumonia (CAP or HAP) 10% no pneumonia		#1 88% #2 97%
Storms [23]	2006–2010	Kaiser VSD group		480–488	Not mentioned No validation				
Rothberg [15]	2007–2010	250,016	Permier’s data base	1ary or secondary to sepsis/resp. failure Chest X ray Abx < 48 h	AIDS patients 0.05% of sample		71% pneumonia		
Sogaard [12]	1995–2007	3516	ICD-10		Yes	Chart review in *N* = 77	Yes		PPV 95%
Current study	2002–2008	6824	ICD-9 Pharmacy	Algorithms^a^ #1480–487 + abx #2480–477 primary or secondary to related codes #2 + abx	Yes (3410 PWH)	Chart review *N* = 397	72% CAP 74% PWH/66% Uninf	NA	#1 76% (PWH77% /Uninf 73%) #2 81% (PWH82%/Uninf75%) #2 + abx 82% (PW H83%/ Uninfected 73%)

*Abx* Antibiotics, *PMC* Patient Management Categories, *LOS* Length of stay

^a^See Table 3 for more details

In recent years, patients admitted for pneumonia are frequently discharged with non-pneumonia ICD-9 codes that reflect greater severity of illness (such as sepsis or respiratory failure) as primary diagnosis [24]. This practice has led to the impression of a decrease in the number of pneumonia admissions while it actually may represent an increase in admissions for severe pneumonia and/or increase coding with higher severity of disease codes [24, 35]. In our study, algorithms that included pneumonia as secondary diagnosis only to selected primary diagnosis (HIV or conditions associated with severe infection) indeed increased the PPV of the ICD-9 pneumonia codes. In addition, allowing common conditions associated with risk for pneumonia among Veterans, such as COPD with acute exacerbation and congestive heart failure as primary diagnosis increased the sample size without markedly affecting the performance of the algorithm. Specifically, allowing the inclusion of chronic bronchitis with acute exacerbation as primary diagnosis improved the PPV in the uninfected group to the highest for this group among all combinations (to 76%), while preserving a high PPV of 82% in the PWH. For almost all of the algorithms evaluated in our study, the PPV was slightly lower in uninfected individuals (range 66–76% compared to 74–83% in PWH) and the difference was statistically significant for some of those that allowed select ICD codes as primary diagnosis.

In addition to ours, the only other study that has specifically evaluated ICD-code performance in PWH hospitalized with pneumonia [12], reported an accuracy of 95% for the ICD codes (vs. 90% in HIV uninfected). This study included a single site sample of 77 PWH (from a multi-site national Danish cohort of 3516 patients with pneumonia), included both CAP and hospital-acquired infections, used a combination of ICD-8 and ICD-10 codes (no ICD-9), and compared it to an accuracy of 90% from a sample of 100 uninfected patients, from a different location and time-period [8]. No statistical analysis was shown for those results.

Our study has several strengths as well as limitations. We are the first to evaluate the performance of EHR data to diagnose CAP in a cohort that compares PWH and uninfected individuals. To allow for maximum flexibility and applicability, we present a series of algorithms to capture CAP admissions from large datasets with or without antimicrobial use. Which EHR-based algorithm to use for a specific study or setting will ultimately depend on the variables available in the data set (not all datasets, including those from Medicare, include antimicrobial prescriptions) and the objectives of the study, knowing that for the most part increases in PPV may come at the cost of a decrease in sample size and missing cases of true CAP. Although composed primarily of male Veterans enrolled in a survey study, our cohort is nationally representative of the aging HIV epidemic and is geographically and racially diverse. Our participants were originally recruited from outpatient clinics, and, in this cohort, most of the PWH and uninfected subjects with pneumonia were admitted from the community; this finding may change as this cohort continues to age.

Our study encompasses years 2002–2008, well within the current ART era and ICD-9 coding practices for community acquired pneumonia [15, 24]. The Veterans Health Administration converted to the International Classification of Diseases, Tenth Revision, Clinical Modification and Procedural Coding System (ICD-10-CM/PCS) in October 2015 [36]. While our data used ICD-9 rather than ICD-10 codes, many database studies encompass time periods utilizing ICD-9 codes and our work can establish the foundation to map validated ICD-9 codes for CAP to new ICD-10 codes [36, 37] among PWH and uninfected patients. Finally, our search strategy was restricted to individuals who had an initial ICD-9 code for CAP selected from a broad set of codes to increase identification of all possible CAP cases. A prior smaller study in Veterans with HIV yielded a sensitivity and specificity of 53 and 95% for ICD-9 codes [13] compared to chart review. Since we did not review the charts of those without a diagnosis of CAP, we are unable to calculate sensitivity and specificity for the different algorithms; it is likely that the proportion of individuals with CAP in our dataset was underestimated. Other approaches to accurately identify CAP in large datasets are currently limited. An algorithm including radiologic findings, potentially retrievable by natural language processing that does not rely on ICD codes, could increase the accurate identification of patients with CAP from EHR.

## Conclusions

It is important to understand the limitations of studies based on ICD codes, a prevalent tool in observational studies encompassing large datasets; however, our study suggest that within the VA system, the capture of CAP diagnosis would not be significantly biased by HIV status, which was a main aim for the study. We present several algorithms for identifying CAP using EHR data with moderately good accuracy. The best algorithm to identify patients with CAP would be the one that best utilizes the available databases and selects the sample that best fits the questions studied. Validated ICD-9 codes can be used in future work to map to ICD-10 codes.

## Supplementary information

**Additional file 1.**

## Data Availability

The datasets used and/or analyzed during the current study are not publicly due to institutional restrictions but are available from the corresponding author on reasonable request.
