# Supplementary material for: Validation for using electronic health records to identify community acquired pneumonia hospitalization among people with and without HIV
Source: Pneumonia (Nathan). 2020 Jul 25;12:6. doi: 10.1186/s41479-020-00068-1 (PMC7382068; doi:10.1186/s41479-020-00068-1)
Supplement: Supplementary file 1 — Additional file 1. [file 41479_2020_68_MOESM1_ESM.pdf]

Validation of Community acquired pneumonia (CAP) and Health care associated pneumonia (HCAP)

Note this form will be used for CAP including *Pneumocystis* pneumonia (PCP) and other community acquired causes of opportunistic pneumonias, as well as HCAP but will not include hospital acquired pneumonia (HAP) or ventilator associated pneumonia (VAP) in patients hospitalized for >48-72 hours.

I. CRITERIA FOR CLINICAL CONFIRMATION

A. Clinical support (answer the following based on review of admission notes):

A1. Compatible clinical findings are present, from at least one of the 6 categories below.

☐ Yes
☐ No (findings denied or are not documented)
☐ Unknown (FLAG FOR ADJUDICTION)

(1) Recent onset or worsening **cough;** new onset of purulent **sputum** or change in character of sputum (gross appearance or by patient report); increased respiratory secretions or increased suctioning requirements; hemoptysis; new onset or worsening of **dyspnea**; new onset or worsening of **tachypnea**; new onset or worsening of **pleuritic chest pain**; **abnormal chest exam** (dullness, bronchial breath sounds, eegophony, rales, crackles, rhonchi, etc.); worsening gas exchange or increased oxygen requirements.

(2) New onset or worsening confusion, agitation, **lethargy**, **delirium,** disorientation, **altered mental status** that is not chronic; new onset or worsening of **fatigue**

(3) **Fever** >38 C (100.4F) or <36 C (96.8F); rigors, chills, night sweats

(4) Leukopenia (<4000 WBC/mm3) or leukocytosis (>12,000 WBC/mm3)

(5) **Nausea, vomiting, diarrhea, abdominal pain**; unable to eat or decreased PO intake; new onset or worsening of anorexia

(6) Other constitutional: **Headache**; **myalgia**; **sore throat**

A2. Patient status prior to admission (select one): Patients transferred from other acute care facilities are included if their initial presentation was for pneumonia. Those admitted for other reasons who developed a pneumonia > 48 hours after hospitalization are excluded; for these patients with hospital acquired pneumonia, check option (1) below and skip to Section II.

☐ Admitted from home / community-dwelling (0)
☐ Transferred from other acute care hospital (1)
☐ Transferred from on-site skilled nursing unit, other nursing home or skilled nursing facility (2)
☐ Transferred from other health care related facility (ie rehabilitation or psychiatric hospital) (3)
☐ Other, Specify: ______________________(4)
☐ Unknown (9)

A3. Indicate if the patient had any of the following risk factors for HCAP present on admission.

- ☐ Discharge from acute care hospital within the last 14 days of current infection (1)
- ☐ Discharge from acute care hospital within the last 90 days of current infection (2)
- ☐ Attended a hemodialysis clinic within 30 days of current infection (3)
- ☐ Received intravenous antibiotics or chemotherapy within last 30 days (4)
- ☐ Received wound care or ventilator care within last 30 days (4)
- ☐ Other, specify: ___________________________
- ☐ None of the above are mentioned in the admission notes / information not available.
- ☐ Unknown (FLAG FOR ADJUDICTION)

B. Radiographic support

B1. Indicate whether radiographic data is from CXR and/or chest CT:

- ☐ CXR
- ☐ CT scan. Use CT obtained within 48 hours of admit to determine findings if no CXR available.

B2. The radiographic findings (select one):

- ☐ Are compatible with pneumonia
- ☐ Are not clearly compatible with pneumonia; uncertain significance (FLAG FOR ADJUDICATION).
- ☐ No acute pulmonary process reported, lungs fields stated to be clear and/or no abnormality noted

Review the radiology report for the chest x-ray obtained **within 48 hours** of admission; if radiology report unavailable, use clinician notes. In cases of discrepancies between radiologist and other clinicians, the radiologist interpretation will be the gold standard.

Terms compatible with pneumonia: air-space or alveolar filling process, airspace disease, bronchogram, bronchopneumonia, consolidation, consolidative process, density, increased interstitial markings, increased lung markings, infection, infectious process, infiltrate, infiltration, infiltrative process, inflammation, inflammatory process, interstitial pneumonia, interstitial process, haziness, opacity, opacification, pneumonia, pneumonic process, pneumonitis, reticulonodular pattern, reticular markings.
Terms for lack of abnormalities: clear lungs; clear lung fields; no acute disease; no infiltrates or other findings noted above.

C. Antibiotic support (Check all that apply): Do not include antimicrobial prophylaxis.

C1. Did the patient receive antibiotics directed against a **bacterial infection** within 48 hours of admission?

- ☐ Yes, antibacterial therapy
- ☐ No
- ☐ Unknown

C2. Did the patient receive antibiotics directed against a **viral, fungal, mycobacterial or parasitic cause of pneumonia** within 5 days of admission? Do not include antimicrobial prophylaxis.

- ☐ Yes, antiviral therapy
- ☐ Yes, anti-pneumocystis therapy
- ☐ Yes, anti-mycobacterial therapy
- ☐ Yes, other anti-fungal therapy
- ☐ Yes, anti-parasitic therapy
- ☐ No
- ☐ Unknown

If C1 or C2 is YES, complete C3:

C3. Were antibiotics directed against the suspected or confirmed pathogen(s) continued for at least 2-3 days, with intention to complete a treatment course? (Check "no" if antibiotics were discontinued because a pathogen was "ruled-out.")

- ☐ Yes
- ☐ No
- ☐ Unknown (FLAG FOR ADJUDICTION)

II. CLINICALLY CONFIRMED DIAGNOSIS BASED ON CHART REVIEW

1. Is this episode of pneumonia **clinically confirmed**? This requires **compatible clinical and radiographic findings and receipt of antimicrobial therapy, where A1=Yes; B1=Yes; and C1 or C2=Yes AND C3=Yes. A confirmed microbiologic diagnosis is not required. Check only one choice. If pneumonia was present on initial admission and nosocomial pneumonia also occurred, select 1st option only.**

- ☐ Yes, this is consistent with pneumonia present on initial admission.
- ☐ No pneumonia is present on admission.
- ☐ This is consistent with pneumonia that developed >48 hours after admission.
- ☐ Uncertain (FLAG FOR ADJUDICATION)

2. Is there **another diagnosis** that accounts for this patient's presentation? Record diagnoses that explain the primary reason for hospitalization**; they may be in addition to or instead of pneumonia**. Mark all that apply; rely primarily on the discharge summary and attending notes for diagnoses if there are discrepancies.

- ☐ Exacerbation of obstructive lung disease: COPD or asthma exacerbation
- ☐ Acute bronchitis, Upper respiratory tract infection (URI) or "Influenza-like" illness
- ☐ Congestive heart failure (CHF), Pulmonary edema, "volume overload"
- ☐ Acute lung injury/acute respiratory distress syndrome (ALI/ARDS)
- ☐ Sepsis / bacteremia
- ☐ Other non-pulmonary infection
- ☐ Lung cancer
- ☐ Pulmonary embolism
- ☐ Other, Specify________________________
- ☐ Unknown / not documented
- ☐ Unclear / FLAG FOR ADJUDICATION
- ☐ No / None

IF THIS IS A CONFIRMED DIAGNOSIS OF PNEUMONIA PLEASE CONTINUE WITH FORM. IF PNEUMONIA IS NOT CONFIRMED OR REQUIRES ADJUDICATION, STOP HERE.

MICROBIOLOGIC DIAGNOSIS OF PNEUMONIA

1. Were any smears obtained? **(Review samples for bacterial culture obtained within initial 48 hours; review samples for other organisms within first 5 days of admission)**

☐ Yes → *GO TO ITEM 2.*

☐ No → *SKIP TO ITEM 3.*

☐ Unknown → *SKIP TO ITEM 3.*

2. Indicate the source of the specimen taken, the findings on smear, and if applicable, the specimen adequacy (only needed if reporting bacterial results). Refer to the list below and enter the letter(s) that correspond to the smear result.

a. Gram positive bacilli
b. Gram positive cocci
c. Gram negative bacilli
d. Gram negative coccobacilli
e. Gram negative cocci
f. Gram stain indeterminate / variable
g. Pneumocystis
h. Cryptococcus
i. Hyphae/pseudohyphae
j. Yeast
k. Acid fast organisms (Ziehl Neelson stain)
l. No organisms seen

Source of specimen:	Findings (insert letter code):	Specimen adequacy: defined as ≥25 PMN/100x field and ≤10 epithelial cells/100x field
a. ☐ Sputum	________________	☐ Adequate<br>☐ Not adequate<br>☐ Unknown/not documented
b. ☐ BAL fluid	________________	
c. ☐ Pleural fluid	________________	
d. ☐ Lung aspirate	________________	
e. ☐ Other, specify:	________________	

3. Were any cultures obtained? (This is for all specimens, including blood and respiratory.)

☐ Yes → *GO TO ITEM 4*

☐ No → *SKIP TO ITEM 8.*

☐ Unknown → *SKIP TO ITEM 8.*

For items 4-6-, refer to the list of organisms below and enter the letter that corresponds to the organism:

4. Was a positive quantitative culture of a protected brush specimen or quantitative BAL culture for a likely pathogen obtained?

☐ Yes → *GO TO Item 5.* ☐ No ↓ *SKIP TO Item 6.*

**Likely bacterial pathogens:**		**Unlikely bacterial pathogens**:
a. Chlamydia<br>b. Coxiella burnetii<br>c. Enterobacter aerogenes<br>d. Escherichia coli	k. Nocardia asteroides<br>l. Pasteurella multocida<br>m. Proteus species<br>n. Pseudomonas aeruginosa	v. Alpha-hemolytic streptococci<br>w. Bacillus species<br>x. Clostridium species<br>y. Coagulase-negative staphylococci
e. Haemophilus influenzae<br>f. Klebsiella pnuemoniae<br>g. Legionella pnuemoniae	o. Rhodococcus equi<br>p. Serratia marcescens<br>q. Staphylococcus aureus – METHCILLIN sensitive	**Other bacteria:**
h. Moraxella catarrhalis	r. Staphylococcus aureus – METHCILLIN resistant	z. Other, specify ______________
i. Mycoplasma pneumoniae<br>j. Neisseria meningitidus	s. Streptococcus pneumoniae<br>t. Streptococcus pyogenes<br>u. Group B streptococci	
**Viruses**<br>A1. Adenovirus<br>A2. Human Metapneumovirus<br>A3. Influenza A (not H1N1)<br>A4. H1N1 Influenza<br>A5. Influenza B<br>A6. Parainfluenza<br>A7. RSV<br>A8. Other, specify.____________	**Fungi**<br>B1. Aspergillus<br>B2. Candida<br>B3. Coccidioides<br>B4. Cryptococcus<br>B5. Histoplasma<br>B6. Other, specify.______________	**Mycobacteria**<br>C1. M. tuberculosis<br>C2. M. avium<br>C3. M. kansasii<br>C4. M. gordonae<br>C5. Other, specify below.<br><br>**Other organism or result**:<br>D1. Specify: _________________<br><br>D2. Normal flora or No Growth

5. Indicate the likely pathogen with a letter: ____

→ *GO TO Item 6 if other cultures also obtained*

6. Indicate the source of each culture with a number and the pathogen(s) isolated with a letter.

Print the names of pathogens not listed in the “Other” column.

Sources:	(1) Blood	(3) Pleural fluid	(5) Lung aspirate
	(2) BAL fluid	(4) Sputum	(6) Other

Source	Pathogen(s) isolated, using letter code above.	Other organisms not listed:	No organism:
a.______	___ | ___ | ___ and/or Other, specify:	_______________	None ☐
b.______	___ | ___ | ___ and/or Other, specify:	_______________	None ☐
c.______	___ | ___ | ___ and/or Other, specify:	_______________	None ☐

7. Were any of the reported sources for specimens in Item 6 “Other” (6)?

☐ Yes →

☐ No ↓

8. Specify the source:

____

STUDY ID __________ Reviewer initials __ __ Date of review __ __ / __ __ / __ __

2. Determine whether a **<u>microbiological</u> diagnosis** for clinically confirmed pneumonia cases is **definite, presumed** or **suspected** for bacterial pneumonia; **definite** or **suspected** for others. Leave blank if the diagnosis was not present; check all that apply.

<u>SUSPECTED</u> microbiologic etiology of any type of infectious pneumonia is present if:
1) Organism is not microbiologically confirmed;
2) Clinical and radiographic presentation was compatible with suspected etiology per the medical record;
3) Patient received antimicrobial therapy directed against the suspected organism with clinical improvement, or if the event of death, antimicrobial therapy against the suspected organism was prescribed.

BACTERIAL PNEUMONIA

A. ☐ <u>Suspected</u> bacterial pneumonia is present

B. ☐ <u>Presumed</u> bacterial pneumonia is present because there is identification of a likely pathogen based on gram stain and culture results from expectorated or induced sputum, or from endotracheal aspirate.

C. ☐ <u>Definite</u> bacterial pneumonia is present because there is isolation of a likely pathogen from:

- ☐ Blood
- ☐ Pleural fluid
- ☐ Bronchoscopic specimen (protected brush at >10^3 cfu/ml or from BAL at > 10^4 cfu/ml).
- ☐ Other normally sterile site (such as urine, cerebrospinal fluid, or other tissue)
- ☐ Histological evidence of bacterial pneumonia in lung tissue (autopsy or biopsy)
- ☐ Detection of Legionella or pneumoccccal antigen in urine or blood
- ☐ Diagnostic serologic findings for Chlamydia, Legionella, Mycoplasma; 4x-rise in titers >3-6 weeks

In <u>definite cases</u>, indicate the bacteria isolated that fulfill the criteria listed above:

**<u>Likely bacterial pathogens:</u>**		**<u>Unlikely bacterial pathogens</u>:**
☐ a. Chlamydia	☐ k. Nocardia asteroides	☐ v. Alpha-hemolytic streptococci
☐ b. Coxiella burnetii	☐ l. Pasteurella multocida	☐ w. Bacillus species
☐ c. Enterobacter aerogenes	☐ m. Proteus species	☐ x. Clostridium species
☐ d. Escherichia coli	☐ n. Pseudomonas aeruginosa	☐ y. Coag-negative staphylococci
☐ e. Haemophilus influenzae	☐ o. Rhodococcus equi	
☐ f. Klebsiella pnuemoniae	☐ p. Serratia marcescens	
☐ g. Legionella pnuemoniae	☐ q. Staphylococcus aureus – METHCILLIN sensitive	**<u>Other bacteria:</u>**
☐ h. Moraxella catarrhalis	☐ r. Staphylococcus aureus – METHCILLIN resistant	☐ z. Other, specify.
☐ i. Mycoplasma pneumoniae	☐ s. Streptococcus pneumoniae	
☐ j. Neisseria meningitidus	☐ t. Streptococcus pyogenes	
	☐ u. Group B streptococci	

B. Pneumocystis Pneumonia (PCP)

☐ Suspected PCP

☐ Definite PCP is present because *Pneumocystis* cysts and/or trophic forms were visualized on microscopic examination of lung derived specimens (e.g., induced sputum, BAL, lung tissue).

C. Fungal pneumonia: Use the table below to indicate definite fungal pneumonia other than PCP.

☐ Suspected fungal pneumonia

☐ Definite fungal pneumonia, with specific etiology noted below, is present based on microscopic, histopathologic or culture results of respiratory-derived specimens; and/or positive serologic or antigen testing.

D. Viral pneumonia: Use the table below to indicate definite viral causes of pneumonia.

☐ Suspected viral pneumonia

☐ Definite viral pneumonia, with specific etiology noted below, is present based on histopathologic or culture results, or PCR-based tests of respiratory-derived specimens.

E. Mycobacterial pulmonary infections: Use the table below to indicate definite mycobacterial causes.

☐ Suspected mycobacterial infection

☐ Definite mycobacterial pulmonary infection, with specific etiology noted below, is present because Mycobacterium species were cultured from lung derived specimens, blood, or extrapulmonary site.

F. Other pneumonia: Use the table below to indicate definite other causes (e.g. parasitic).

☐ Suspected other infection

☐ Definite other infectious cause of pneumonia, with specific etiology as noted below, is present based on microscopic, histopathologic or culture results of respiratory-derived specimens; and/or positive serologic or antigen testing

G. Indicate all other organisms that were definite cause(s) of pneumonia above in C-F:

**Viruses**	**Fungi**	**Mycobacteria**
☐ A1. Adenovirus	☐ B1. Aspergillus	☐ C1. M. tuberculosis
☐ A2. Human Metapneumovirus	☐ B2. Candida	☐ C2. M. avium
☐ A3. Influenza A (not H1N1)	☐ B3. Coccidioides	☐ C3. M. kansasii
☐ A4. H1N1 Influenza	☐ B4. Cryptococcus	☐ C4. M. gordonae
☐ A5. Influenza B	☐ B5. Histoplasma	☐ C5. Other, specify below.
☐ A6. Parainfluenza	☐ B6. Other, specify.	
☐ A7. RSV		**Other organism not specified**:
☐ A8. Other, specify.		☐ D1. Specify

H. Microbiologic diagnosis(-es) unclear; chart requires adjudication. ☐ Yes ☐ No

STUDY ID _________ Reviewer initials __ __ Date of review __ __ / __ __ / __ __
